# A maternal high-fat, high-sucrose diet has sex-specific effects on fetal glucocorticoids with little consequence for offspring metabolism and voluntary locomotor activity in mice

**DOI:** 10.1371/journal.pone.0174030

**Published:** 2017-03-16

**Authors:** Eunice H. Chin, Kim L. Schmidt, Kaitlyn M. Martel, Chi Kin Wong, Jordan E. Hamden, William T. Gibson, Kiran K. Soma, Julian K. Christians

**Affiliations:** 1 Department of Biological Sciences, Simon Fraser University, Burnaby, BC, Canada; 2 Department of Psychology, University of British Columbia, Vancouver, BC, Canada; 3 Department of Medical Genetics, University of British Columbia and BC Children's Hospital, Vancouver, BC, Canada; 4 Department of Zoology, University of British Columbia, Vancouver, BC, Canada; Xavier Bichat Medical School, INSERM-CNRS - Université Paris Diderot, FRANCE

## Abstract

Maternal overnutrition and obesity during pregnancy can have long-term effects on offspring physiology and behaviour. These developmental programming effects may be mediated by fetal exposure to glucocorticoids, which is regulated in part by placental 11β-hydroxysteroid dehydrogenase (11β-HSD) type 1 and 2. We tested whether a maternal high-fat, high-sucrose diet would alter expression of placental 11β-HSD1 and 2, thereby increasing fetal exposure to maternal glucocorticoids, with downstream effects on offspring physiology and behaviour. C57BL/6J mice were fed a high-fat, high-sucrose (HFHS) diet or a nutrient-matched low-fat, no-sucrose control diet prior to and during pregnancy and lactation. At day 17 of gestation, HFHS dams had ~20% lower circulating corticosterone levels than controls. Furthermore, there was a significant interaction between maternal diet and fetal sex for circulating corticosterone levels in the fetuses, whereby HFHS males tended to have higher corticosterone than control males, with no effect in female fetuses. However, placental 11β-HSD1 or 11β-HSD2 expression did not differ between diets or show an interaction between diet and sex. To assess potential long-term consequences of this sex-specific effect on fetal corticosterone, we studied locomotor activity and metabolic traits in adult offspring. Despite a sex-specific effect of maternal diet on fetal glucocorticoids, there was little evidence of sex-specific effects on offspring physiology or behaviour, although HFHS offspring of both sexes had higher circulating corticosterone at 9 weeks of age. Our results suggest the existence of as yet unknown mechanisms that mitigate the effects of altered glucocorticoid exposure early in development, making offspring resilient to the potentially negative effects of a HFHS maternal diet.

## Introduction

Adult physiology and behaviour are influenced by early life environment, including maternal diet and stress during pregnancy [1–5]. For example, maternal overnutrition and/or obesity during pregnancy can have long-term effects on metabolic and cardiovascular traits in adult offspring [1,4]. Maternal consumption of a high-fat diet and/or obesity also affect offspring mental health in humans as well as behaviour in animal models [2,3,6,7], including voluntary locomotor activity [8,9]. Similarly, early postnatal nutrition influences voluntary physical activity in adulthood [10]. However, whether effects of early life environment on metabolic and cardiovascular traits are caused, at least in part, by programming effects on physical activity has received little attention [11].

Developmental programming of adult physiology and behaviour may be mediated by fetal exposure to glucocorticoids [12,13]. The transfer of glucocorticoids from maternal to fetal circulation is regulated in part by 11β-hydroxysteroid dehydrogenase type 2 (11β-HSD2) [13]. This enzyme, which is highly expressed in the placenta, inactivates glucocorticoids into metabolites (corticosterone to 11-dehydrocorticosterone in rodents and cortisol to cortisone in humans) that have lower affinity for their cognate receptors [13]. Conversely, 11β-HSD1, also present in the placenta, regenerates glucocorticoids from metabolites, thereby restoring activity [14]. Placental 11β-HSD2 activity is generally reduced by maternal malnutrition [13,15,16]. However, while a low protein diet decreased 11β-HSD2 activity near the end of pregnancy, it had the opposite effect earlier in pregnancy [17]. Similarly, maternal stress generally reduces placental 11β-HSD2 activity [5,18,19], although some studies have found the opposite pattern [5]. The effects of maternal overnutrition and/or obesity on placental 11β-HSD2 activity have received less study [20], although a high-fat diet decreases placental 11β-HSD2 expression and enzymatic activity in mice [21,22].

We hypothesized that a maternal high-fat, high-sucrose diet would alter expression of placental 11β-HSD1 and 2, thereby increasing fetal exposure to maternal glucocorticoids, with downstream effects on offspring voluntary energy expenditure. We tested this hypothesis in a mouse model of maternal obesity by measuring the voluntary locomotor activity of adult offspring [8,9]. We also assessed the effects of the diet manipulation on pregnancy and other aspects of offspring programming frequently measured in other studies. In particular, we measured placental phosphorylation of protein kinase B (Akt) as a measure of insulin signaling [23–25] and placental expression of nutrient transporters [23,26], as well as metabolic traits of the offspring including food intake, energy expenditure and glucose tolerance [8]. Because leptin can affect 11β-HSD2 activity [27] and because glucocorticoids may regulate leptin expression [28], we also measured leptin in the maternal and fetal circulations.

## Methods

All work was carried out in accordance with the guidelines of the Canadian Council on Animal Care and approved by the SFU University Animal Care Committee and the UBC Animal Care Committee (protocols 1094 and A13-0006, respectively). C57BL/6J mice were purchased from the Jackson Laboratory (stock # 664) and were group-housed in individually ventilated cages (50 air changes/hour; max. 5 mice per cage) with Enrich-o'Cobs bedding (Andersons Lab Bedding, Maumee, OH) on a 12:12 hour light:dark cycle, at constant temperature (21 ± 1°C), 50% humidity, with water and food available ad libitum. At 8–11 weeks of age, females were placed on either a high-fat, high-sucrose diet (HFHS; 45% kcal fat, 35% carbohydrate (including 17% kcal sucrose), 20% kcal protein, 4.73 kcal/g, D12451, Research Diets, New Brunswick, NJ) or a nutrient-matched low-fat, no-sucrose control diet (CON; 10% kcal fat, 70% kcal carbohydrate (corn starch and maltodextrin), 20% kcal protein, 3.85 kcal/g, D12450K, Research Diets).

One group of females was euthanized during pregnancy. These females were kept on the experimental diets for 13 weeks prior to being paired with a male for one night, checked for vaginal plugs, and kept on their experimental diet until euthanized at day 17 of pregnancy (where the day the vaginal plug was observed = day 0). If no vaginal plug was observed and/or if female weight had not increased by ~1g one week after mating, females were paired again. Because not all females became pregnant when first paired, females euthanized during pregnancy had been on the experimental diet for 13–17 weeks at the start of pregnancy.

Pregnant females were euthanized by cervical dislocation within 2.5 minutes of moving their home cage, and immediately blood sampled by cardiac puncture. Fetal blood was collected into heparinized tubes following decapitation as quickly as possible after euthanization of the mother. Placentae were either stored in RNAlater (Ambion, Foster City, CA), or placed on dry ice and then stored at -80°C for protein extraction. Fetal sex was determined by PCR [29]. In this experiment, 19 HFHS and 20 CON females were paired, but 7 of each diet did not become pregnant within 17 weeks on the experimental diet, and 1 CON female carried only 2 fetuses and so was excluded from analyses.

To assess the long-term consequences of the maternal diet on the offspring, another group of females was allowed to deliver and rear their pups, remaining on the experimental diet throughout pregnancy and lactation. Because a number of females lost first litters to cannibalism or neglect (described below), only the offspring of second litters were studied. To match the timing of the second pregnancy to that of the females euthanized during pregnancy (described above), females were kept on the experimental diets for 9–10 weeks prior to being paired with a male, such that their first pregnancy started after 10 weeks on the experimental diet, and their second pregnancy started after ~ 13 weeks on the experimental diet. Offspring were weaned at three weeks of age and maintained on breeding chow (Prolab RMH 2000 5P06, 23% kcal fat, LabDiet, St. Louis, MO) until 6 weeks of age, when they were gradually switched to normal chow (5001, 13.5% kcal fat, LabDiet), i.e., remaining breeding chow was left in the food hopper, but was topped up with normal chow.

Offspring of two cohorts were studied, with some improvements in experimental protocol for the second cohort. In the first cohort, some females were nursing their first litter while pregnant with their second litter, whereas in the second cohort the male was removed before the birth of the first litter, and not returned until a week after the first litter had been euthanized (soon after birth). In the first cohort, litter size was not standardized, whereas in the second cohort, litter size was standardized at birth to a maximum of 6. In the first cohort, 6 females per diet were paired with males, but one of the HFHS females cannibalized her first and second litters and so was not included. In the second cohort, 10 females per diet were paired, but one CON female cannibalized her first and second litters, one HFHS female died during the birth of her first litter and one HFHS female was never observed to produce a litter.

### Glucose tolerance

Glucose tolerance was measured in a subset of dams (N = 5/ diet) three days before pairing, and in offspring at 15–18 weeks of age. Glucose tolerance tests were performed after a 5 hour fast on unanesthetized animals [30,31]. Mice were given an intraperitoneal injection of 20% glucose at a dose of 2 g D-glucose/kg body weight [32], and blood sampled from the saphenous vein at 0, 15, 30, 60 and 120 minutes after injection. Blood glucose levels were measured using an AlphaTRAK 2 glucometer (Abbott, Illinois). A blood sample taken immediately prior to glucose challenge was frozen for measurement of plasma triglycerides. All injections were performed between 12 and 1 pm.

### Voluntary activity

Voluntary locomotor activity during the dark phase was measured in offspring at 16–19 weeks of age using angled running wheels (15.5 cm diameter) that were monitored wirelessly (Wheel Manager software, Med Associates Inc., VT, USA). In the first cohort, offspring were provided with a running wheel while group-housed to allow habituation, and three days later were single housed for activity measurements throughout the subsequent four nights. In the second cohort, mice were single-housed upon addition of the running wheel, habituated to the running wheel for three days, followed by four nights of activity measurement.

### Metabolic and body composition phenotyping

Food and water intake, energy expenditure, oxygen consumption, carbon dioxide production, respiratory exchange ratio and spontaneous physical activity were measured in mice housed individually in metabolic cages, as previously described [33]. Whole animal body composition was measured by quantitative magnetic resonance (QMR) analysis, as previously described [33]. Body composition was measured post-mortem for dams and while alive for adult offspring.

### Serum analytes

Kits were used to measure circulating levels of corticosterone (07120103, MP Biomedicals, as described in [34]), leptin (90030, Crystal Chem), IL-6 (M6000B, R&D Systems) and triglycerides (Sigma-Aldrich).

### Quantitative PCR

Tissue was homogenized in buffer RLT using pestles and Qiashredders, and total RNA was extracted using the RNeasy Mini kit (Qiagen, Ontario, Canada). RNA concentration was determined using a Nanodrop spectrophotometer (Thermo Fischer Scientific Inc., Waltham, MA). A reference sample was prepared by combining samples and was included in every assay to account for variation between assays. In addition to the mRNA levels of *Hsd11b1* and *Hsd11b2*, we also measured *Slc38a2*, *Slc27a4*, *Slc2a1*, genes encoding neutral amino acid, long-chain fatty acid and glucose transporters, respectively, expressed in the placenta [23,26,35] and *β-actin* as a housekeeping gene. Primer sequences are shown in Table 1. The qScript 1-step SYBR Green qRT-PCR kit (Quanta Biosciences Inc. Gaithersburg, MD) was used to reverse-transcribe and amplify each sample for 40 cycles. At each cycle, the amount of fluorescence was quantified using a miniOpticon (Bio-Rad, Hercules, CA), and the cycle at which the signal rose above a fixed threshold (Ct) was determined. Each sample was analysed in duplicate. We used the method of Pfaffl [36] to calculate mRNA expression levels relative to the reference sample, e.g., a value of 1.5 indicates a sample has 50% more of a particular transcript than the reference sample, correcting for *β-actin*.

**Table 1:** Primer sequences used for quantitative PCR.

Gene	Forward	Reverse
*β-actin*	CAGGTCATCACTATTGGCAACGAG	ACGGATGTCAACGTCACACTTCAT
*Hsd11b1*	GAGGAAGGTCTCCAGAAGGTA	ATGTCCAGTCCGCCCAT
*Hsd11b2*	GGCTGGATCGCGTTGTC	CGTGAAGCCCATGGCAT
*Slc38a2*	CATGGCTAATACTGGAATTGCTC	CCTTATGTCCCAACTGTTCGTA
*Slc27a4*	TTCTTGCCTGAGCTGCAC	CCGAGCATCCAGATAGAACAG
*Slc2a1*	AGTTCGGCTATAACACTGGTG	GTGGTGAGTGTGGTGGATG

### Western blotting

Protein extraction and Western blotting were performed as described previously [37]. Membranes were incubated overnight at 4°C with a primary antibody solution containing antibodies against actin and one protein of interest. Primary antibodies were as follows: actin (CLT9001, Cedarlane), Akt (9272S, New England Biolabs), phospho-Akt (Ser473; 4060S, New England Biolabs), 11β-HSD1 (AB39364, Cedarlane), 11β-HSD2 (AB80317, Cedarlane). Membranes were visualized using the Odyssey infrared imaging system (Li-Cor Biosciences, Lincoln, NE) which allowed simultaneous quantification of the protein of interest and actin using secondary antibodies that fluoresced at different wavelengths.

### Statistical analyses

All statistical analyses were performed using general linear models (proc GLM) or repeated measures analyses (proc MIXED) in SAS, Version 9.3 (SAS Institute Inc., Cary, NC). Repeated measures analyses were used for all placental, fetal and offspring traits where there were multiple offspring per dam (with dam as a random factor), since the dam was the unit of replication. Models initially included a maternal diet by offspring sex interaction term to test for sex-specific effects [38] and this term was removed if not significant (P < 0.05). Where a diet by sex interaction was significant, the effect in males and females was tested using the ESTIMATE statement.

## Results

### Maternal traits

**Fig 1:**
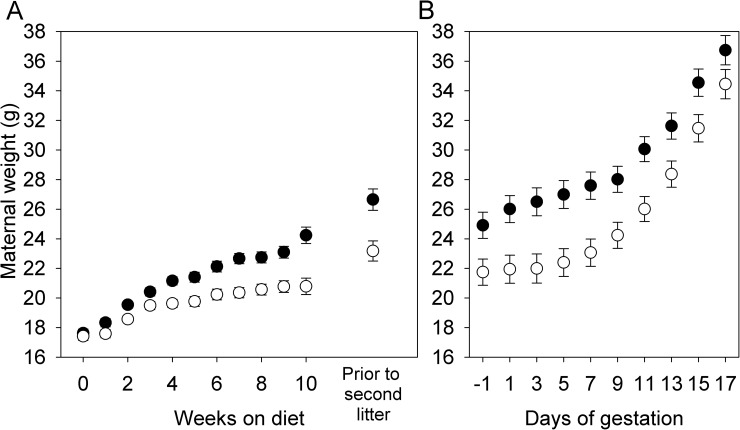
Effect of maternal diet on weight gain prior to and during pregnancy. (A) Weight gain prior to pregnancy (N = 6 per diet in first cohort and 30 per diet in second cohort) and (B) during pregnancy (N = 12 per diet) in females fed a high-fat, high-sucrose (HFHS, solid symbols) or low-fat, no-sucrose (CON, open symbols) diet. Values are least squares means ± standard error. Analyses of pre-pregnancy weight gain included effects of cohort; the diet by cohort interaction term was not significant at any time point and so was removed from the models.

**Fig 2:**
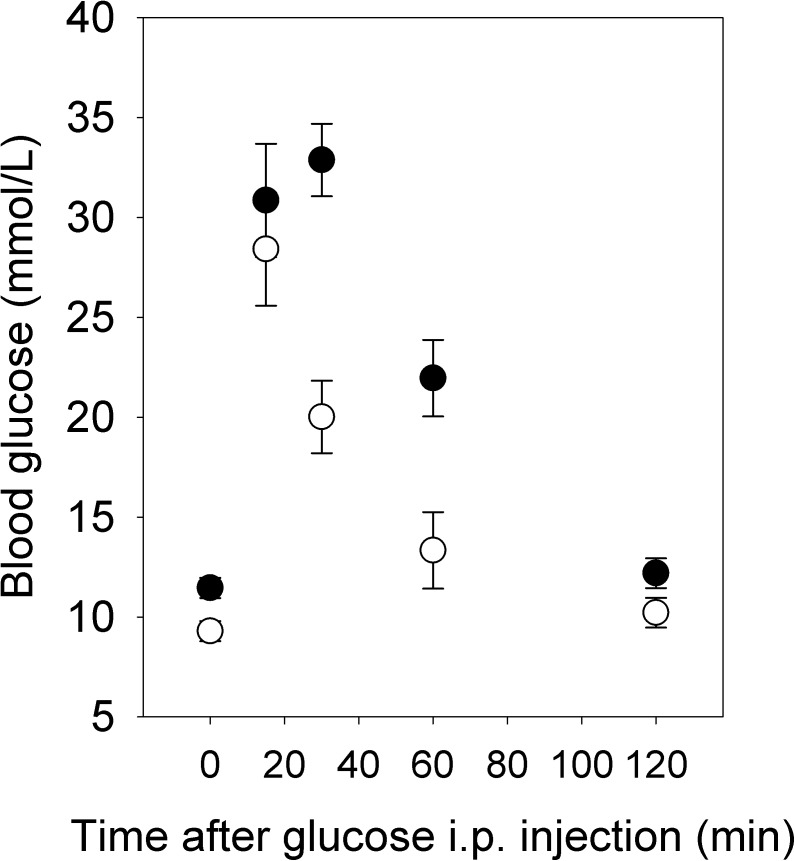
Glucose tolerance prior to mating. Values for HFHS females (solid symbols) and CON females (open symbols) are least squares means ± standard error (N = 5 per diet).

**Fig 3:**
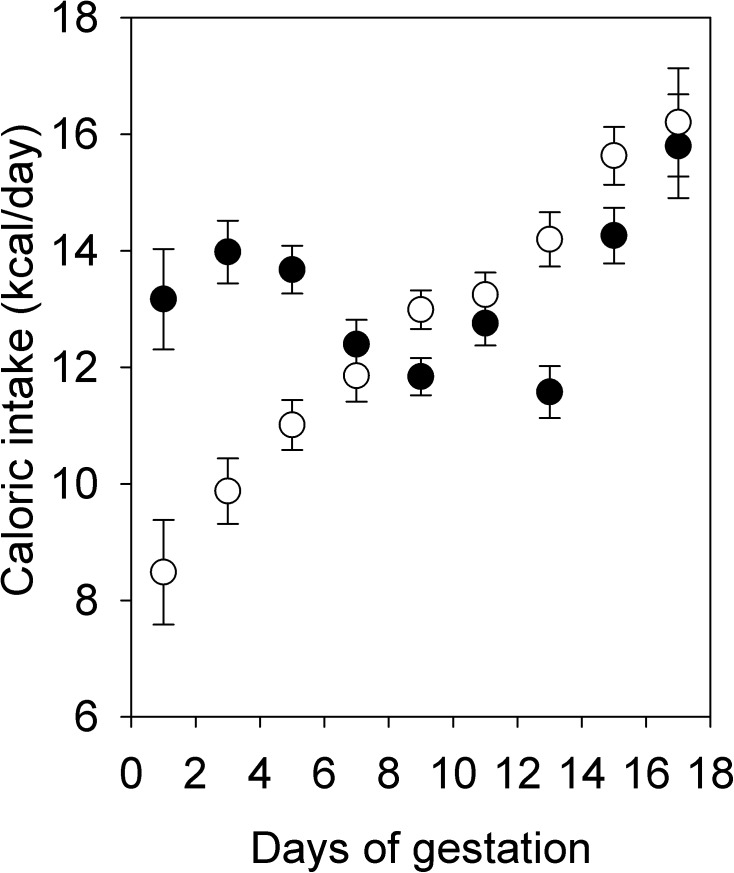
Caloric intake during pregnancy. Values for HFHS dams (solid symbols; N = 12) and CON dams (open symbols; N = 11 due to missing data for one mouse) are least squares means ± standard error.

As expected, females on a HFHS diet gained more weight than females on the CON diet, such that they were significantly heavier at the time of first pairing (F_1,15_ = 12.32, P = 0.0032) and were ~15% heavier prior to becoming pregnant with their second litter (Fig 1A). Three days before mating for the first litter, HFHS females had higher fasting blood glucose levels (F_1,8_ = 9.19, P = 0.02; Fig 2) and reduced glucose tolerance measured as the area under the curve (AUC, F_1,8_ = 16.45, P < 0.01) or the positive incremental area under the curve (i.e., the area under the curve, but above the baseline level, piAUC, F_1,8_ = 13.81, P < 0.01; Fig 2). However, fasting triglyceride levels did not differ between diets (HFHS: 0.48 ± 0.06 mmol/L; CON: 0.39 ± 0.05 mmol/L; F_1,7_ = 1.32, P = 0.29). Among females paired with males for only one night, such that the timing of pregnancy was known, HFHS females weighed more at the beginning of pregnancy (F_1,22_ = 6.37, P = 0.02; Fig 1B), but CON females tended to gain more weight between pairing and day 17 of gestation (F_1,21_ = 3.11, P = 0.09, controlling for number of conceptuses, F_1,21_ = 21.33, P < 0.0001). As a result, although HFHS females tended to be heavier at day 17 of gestation (Fig 1B), this difference was not significant (F_1,22_ = 2.69, P = 0.12). HFHS females euthanized at day 17 of gestation had greater fat mass, but not greater lean mass, than CON females, as measured by QMR (Table 2). Consistent with weight gain, HFHS females consumed more calories at the beginning of pregnancy, whereas CON females caught up by day 17 (Fig 3). Because the two diets were equal with respect to the proportion of calories obtained from protein (20%), protein consumption through pregnancy paralleled caloric consumption.

**Table 2:** Physiological and reproductive traits among females euthanized at day 17 of gestation.

	HFHS	CON	F	DF	P	Terms in model in addition to diet
N	12	12				
Fat mass (g)	8.1 ± 0.6	5.6 ± 0.6	9.55	1, 22	0.0053	
Lean mass (g)	19.8 ± 0.2	19.9 ± 0.2	0.32	1, 22	0.58	
Serum corticosterone (ng/mL)	751 ± 36	937 ± 36	13.20	1, 22	0.0015	
Serum leptin (ng/mL)	174 ± 24	107 ± 23	4.17	1,21	0.0540	
Serum IL-6 (pg/mL)	6.8 ± 2.5	2.9 ± 2.7	1.16	1,7^1^	0.32	
Number of conceptuses	7.5 ± 0.4	7.2 ± 0.4	0.29	1, 22	0.60	
Fetal sex ratio (% males)	47 ± 6	53 ± 6	0.56	1, 22	0.46	
Fetal weight (mg)^2^	809 ± 37	852 ± 37	0.70	1, 21	0.41	Fetal sex: F_1,22_ = 8.14, P = 0.009^6^; Number of conceptuses: F_1,21_ = 3.47, P = 0.08^5^
Placental weight (mg)^2^	111 ± 3	112 ± 3	0.00	1, 21	0.95	Fetal sex: F_1,22_ = 26.39, P < 0.0001^6^; Number of conceptuses: F_1,21_ = 3.29, P = 0.08^5^
Fetal: placental weight ratio^2^	7.5 ± 0.4	7.7 ± 0.4	0.15	1, 21	0.70	Fetal sex: F_1,22_ = 5.90, P = 0.02; Number of conceptuses: F_1,21_ = 0.36, P = 0.55
Fetal leptin (ng/mL)^2^	0.9 ± 0.3	0.5 ± 0.3	0.96	1, 34^3^	0.33	Fetal sex: F_1,34_ = 0.02, P = 0.90
Placental pAkt (arbitrary units)^4^	1.02 ± 0.03	1.01 ± 0.03	0.22	1,22	0.65	Fetal sex: F_1,12_ = 7.67, P = 0.02^7^
Placental total Akt (arbitrary units)^4^	1.10 ± 0.05	0.94 ± 0.05	4.91	1, 22	0.04	Fetal sex: F_1,12_ = 0.10, P = 0.76
pAkt: total Akt ratio	0.95 ± 0.04	1.11 ± 0.05	6.31	1, 22	0.02	Fetal sex: F_1,12_ = 3.63, P = 0.08
*Slc38a2* mRNA	1.4 ± 1.4	1.2 ± 1.4	0.18	1, 19	0.67	Fetal sex: F_1,6_ = 3.54, P = 0.11
*Slc27a4* mRNA	1.1 ± 1.9	0.6 ± 1.9	0.48	1, 20	0.50	Fetal sex: F_1,7_ = 8.89, P = 0.02^7^
*Slc2a1* mRNA	0.6 ± 1.3	1.2 ± 1.4	2.51	1, 20	0.13	Fetal sex: F_1,7_ = 0.31, P = 0.60
Placental IL-6 protein (pg/ ±g total protein)	0.28 ± 0.03	0.31 ± 0.03	0.59	1, 22	0.45	Fetal sex: F_1,15_ = 0.16, P = 0.69

^1^ Plasma was pooled between dams and therefore the sample size was reduced.

^2^ The interaction between sex and diet was initially included in the model, but was not significant and so was removed.

^3^ Plasma was pooled between fetuses of different dams for measurement of fetal leptin, and this analysis did not use repeated measures analysis.

^4^ Corrected for actin.

^5^ The relationship between the number of conceptuses and fetal and placental weight was negative.

^6^ Male conceptuses were heavier and had heavier placentas.

^7^ Male conceptuses had lower values.

Among females euthanized at day 17 of gestation, HFHS females had 20% lower circulating corticosterone levels and 63% higher leptin levels than CON females, although the latter difference was marginally non-significant (P = 0.054; Table 2). Circulating IL-6, a measure of systemic inflammation, did not differ between diets (Table 2).

### Fetal and placental traits

There was no difference between diets in the number of conceptuses at day 17 or in the fetal sex ratio (Table 2). There was no effect of diet on fetal weight, placental weight or the fetal-to-placental weight ratio, including the number of conceptuses as a covariate in the models (Table 2).

**Fig 4:**
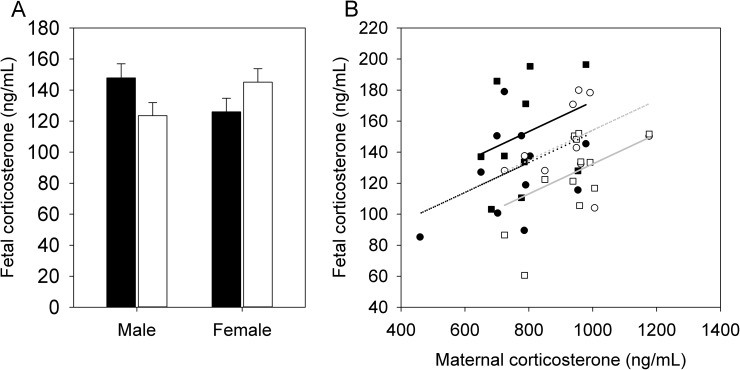
Fetal corticosterone levels. (A) Fetal (day 17) corticosterone levels in HFHS dams (solid bars; N = 12 dams) and CON dams (open bars; N = 12 dams). Values are least squares means ± standard error from a repeated measures analysis including effects of diet, fetal sex, and the diet by fetal sex interaction and dam as the random subject. (B) Correlation between fetal and maternal corticosterone in HFHS (solid symbols, black lines) and CON (open symbols, gray lines) male (squares, solid lines) and female (circles, dotted lines) fetuses. The regression lines have a shared slope because there was no significant interaction between maternal corticosterone and fetal sex, or between maternal corticosterone and maternal diet; there was only an interaction between fetal sex and diet.

There was no interaction between maternal diet and fetal sex (i.e., no difference in the effect of maternal diet between the sexes) and no significant effects of maternal diet or fetal sex on fetal circulating leptin levels (Table 2). However, there was a significant interaction between maternal diet and fetal sex on fetal circulating corticosterone levels (F_1,18_ = 8.59, P = 0.009), whereby HFHS male fetuses tended to have higher plasma corticosterone than CON male fetuses (t_18_ = 1.96, P = 0.07), with no effect of maternal diet in female fetuses (t_18_ = -1.55, P = 0.14) (Fig 4A). Among HFHS fetuses, males tended to have higher corticosterone than females (t_18_ = -2.03, P = 0.06), whereas the opposite pattern occurred in CON fetuses (t_18_ = 2.12, P = 0.05). When maternal corticosterone was added to the model as a covariate, maternal corticosterone was significantly correlated with fetal corticosterone (F_1,21_ = 6.37, P = 0.02), and the interaction between maternal diet and fetal sex remained significant for fetal corticosterone (F_1,18_ = 8.35, P = 0.01), again with HFHS male fetuses having higher corticosterone than CON male fetuses (t_18_ = 3.08, P = 0.007), with no effect in female fetuses (t_18_ = -0.11, P = 0.91) (Fig 4B).

**Fig 5:**
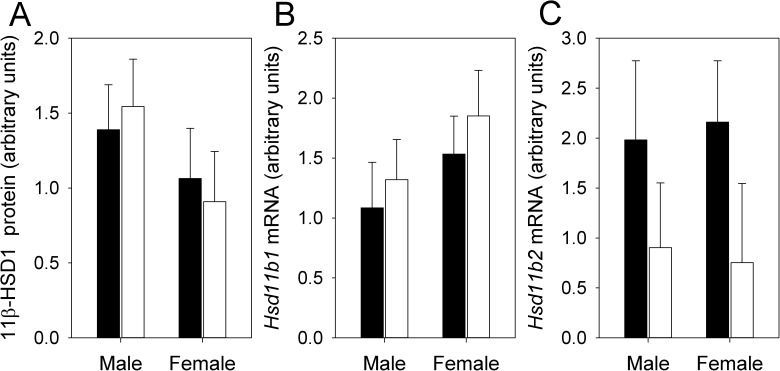
Placental 11β-HSD expression. (A) 11β-HSD1 protein, (B) Hsd11b1 mRNA and (C) Hsd11b2 mRNA from HFHS dams (solid bars; N = 12 dams) and CON dams (open bars; N = 12 dams). Values are least squares means ± standard error from a repeated measures analysis including effects of diet, fetal sex, and the diet by fetal sex interaction, with dam as the random subject.

While HFHS dams had lower circulating corticosterone than CON, their male fetuses had higher corticosterone than those of CON dams. This contrasting pattern suggested sex-specific changes in 11β-HSD1 and/or 11β-HSD2 expression. However, there was no significant interaction between maternal diet and fetal sex, and no significant effects of maternal diet or fetal sex, on 11β-HSD1 or 11β-HSD2 mRNA levels, or 11β-HSD1 protein levels (P > 0.1 in all cases) (Fig 5). We could not detect 11β-HSD2 by Western blotting, consistent with much higher quantitative PCR Ct values (i.e, lower expression) for 11β-HSD2 than for 11β-HSD1, as well as previous reports that 11β-HSD2 expression decreases towards the end of gestation [14,39].

As a measure of insulin signaling and Akt activity, we measured placental protein levels of phosphorylated Akt (Akt-S473) and total Akt by Western blotting. There was no significant interaction between maternal diet and fetal sex on either trait, or on the ratio of phosphorylated to total Akt. There was no effect of maternal diet on the levels of phosphorylated Akt, but HFHS placentae had significantly higher total Akt, and as a result had a significantly lower ratio of phosphorylated to total Akt (Table 2). There was no effect of maternal diet on the mRNA levels of genes encoding amino acid, fatty acid or glucose transporters (Table 2). We measured placental IL-6 by ELISA to assess inflammation, but found no significant interaction between maternal diet and fetal sex, and no significant effects of maternal diet or fetal sex (Table 2).

### Offspring perinatal traits

Of the first litters that were not euthanized immediately after birth, all pups had died by the next day in 6 of 9 HFHS litters, whereas only 1 of 12 first CON litters were completely lost (Fisher’s Exact Test P = 0.02). As a result, subsequent first litters from the second cohort were euthanized on the day of birth.

The number of pups at birth in the second litters was significantly lower in HFHS dams in the first cohort, but not in the second cohort (Table 3). When the two cohorts were combined, the diet by cohort interaction was marginally non-significant (F_1,24_ = 4.06, P = 0.06). Conversely, birth weight did not differ between diets in either cohort (Table 3). There was no difference in the number of days between mating and birth in either cohort (Table 3).

**Table 3:** Peri- and postnatal traits of offspring.

	HFHS	CON	F	DF	P	Terms in model in addition to diet
N						
First cohort	5	6				
Second cohort	8	9				
Litter size at birth						
First cohort	6.6 ± 0.4	8.3 ± 0.3	11.29	1, 9	0.008^1^	
Second cohort	7.8 ± 0.4	7.9 ± 0.4	0.07	1, 15	0.80	
Birth weight (g)						
First cohort	1.31 ± 0.03	1.25 ± 0.02	2.04	1, 8	0.19	Litter size: F_1,8_ = 1.54, P = 0.25
Second cohort	1.31 ± 0.02	1.31 ± 0.02	0.04	1,14	0.84	Litter size: F_1,14_ = 6.77, P = 0.02
Time between mating and birth (days)						
First cohort	23.0 ± 1.4	22.8 ± 1.3	0.01	1, 9	0.93	
Second cohort	21.8 ± 0.4	21.8 ± 0.4	0.00	1, 15	0.96	
Food consumption at 5–6 weeks (g/ day/ mouse)^2^						
First cohort	3.77 ± 0.12	3.41 ± 0.12	4.44	1, 8	0.07	Sex: F_1,9_ = 9.08, P = 0.01; Week (5 vs. 6): F_1,9_ = 3.00, P = 0.12
Second cohort	4.46 ± 0.11	4.12 ± 0.10	5.36	1, 15	0.04	Sex: F_1,12_ = 1.12, P = 0.31; Week (5 vs. 6): F_1,13_ = 2.39, P = 0.15
Voluntary locomotor activity (1000s of wheel rotations)^2^						
First cohort	15.9 ± 2.0	20.7 ± 1.9	3.11	1, 9	0.11	Sex: F_1,10_ = 56.4, P < 0.0001^5^; Night: F_3,30_ = 9.4, P = 0.0002
Second cohort	16.9 ± 1.1	16.5 ± 1.1	0.08	1, 15	0.78	Sex: F_1,15_ = 65.2, P < 0.0001^5^; Night: F_3,48_ = 4.9, P = 0.005
Traits measured in second cohort only						
Fasting triglyceride at 15–18 weeks (mmol/L)^2^	0.23 ± 0.02	0.24 ± 0.02	0.16	1, 15	0.69	Sex: F_1,15_ = 5.97, P = 0.03^4^
Preference for HFHS diet at 17–22 weeks^3^	0.941 ± 0.008	0.952 ± 0.007	1.06	1, 15	0.32	Sex: F_1,15_ = 6.60, P = 0.02^4^
Metabolic traits at 18–23 weeks						
Food intake (g/hour)^2^						
Dark phase	0.19 ± 0.02	0.17 ± 0.02	0.40	1, 13	0.54	Sex: F_1,6_ = 2.90, P = 0.14
Light phase	0.06 ± 0.01	0.05 ± 0.01	0.02	1, 13	0.88	Sex: F_1,6_ = 0.88, P = 0.38
Water intake (mL/hour)^2^						
Dark phase	0.29 ± 0.02	0.29 ± 0.02	0.00	1, 13	0.99	Sex: F_1,6_ = 12.37, P = 0.01^4^
Light phase	0.09 ± 0.01	0.08 ± 0.01	0.53	1, 13	0.48	Sex: F_1,6_ = 5.21, P = 0.06^4^
Energy expenditure (kcal/hour)^2^						
Dark phase	0.36 ± 0.02	0.37 ± 0.01	0.16	1, 13	0.70	Sex: F_1,6_ = 1.72, P = 0.24
Light phase	0.26 ± 0.01	0.27 ± 0.01	1.05	1, 13	0.32	Sex: F_1,6_ = 0.08, P = 0.78
Spontaneous physical activity (counts/hour)^2^						
Dark phase	2512 ± 883	3193 ± 826	0.35	1, 13	0.57	Sex: F_1,6_ = 3.93, P = 0.09
Light phase	527 ± 160	815 ± 150	1.86	1, 13	0.20	Sex: F_1,6_ = 10.03, P = 0.02
Oxygen consumption (mL/hour)^2^						
Dark phase	73 ± 3	74 ± 3	0.18	1, 13	0.68	Sex: F_1,6_ = 1.60, P = 0.25
Light phase	53 ± 2	55 ± 2	1.16	1, 13	0.30	Sex: F_1,6_ = 0.15, P = 0.72
Carbon dioxide production (mL/hour)^2^						
Dark phase	67 ± 4	68 ± 3	0.07	1, 13	0.80	Sex: F_1,6_ = 3.67, P = 0.10
Light phase	46 ± 2	48 ± 2	0.50	1, 13	0.49	Sex: F_1,6_ = 0.00, P = 0.98
Respiratory exchange ratio^2^						
Dark phase	0.91 ± 0.02	0.91 ± 0.02	0.05	1, 13	0.82	Sex: F_1,6_ = 13.69, P = 0.01^4^
Light phase	0.87 ± 0.01	0.86 ± 0.01	0.24	1, 13	0.63	Sex: F_1,6_ = 0.41, P = 0.54
Fat mass (g)^2^	5.2 ± 0.2	5.1 ± 0.2	0.01	1, 15	0.92	Sex: F_1,15_ = 37.98, P < 0.0001^4^; Age: F_1,73_ = 16.69, P < 0.0001
Lean mass (g)^2^	21.3 ± 0.3	21.2 ± 0.2	0.02	1, 15	0.90	Sex: F_1,15_ = 244.84, P < 0.0001^4^; Age: F_1,73_ = 0.58, P = 0.45

^1^ Also significant by non-parametric Wilcoxon and Kruskal-Wallis tests.

^2^ Food consumption at 5–6 weeks was measured in 17 mice from 5 HFHS dams and 22 mice from 5 CON dams in the first cohort, and 32 mice from 8 HFHS dams and 41 mice from 9 CON dams in the second cohort. Voluntary locomotor activity was measured in 27 mice from 5 HFHS dams and 29 mice from 6 CON dams in the first cohort, and 43 mice from 8 HFHS dams and 44 mice from 9 CON dams in the second cohort. Food intake, water intake, energy expenditure, spontaneous physical activity, oxygen consumption, carbon dioxide production and the respiratory exchange ratio were measured in a subset of 11 HFHS mice (from 7 dams) and 12 CON mice (from 8 dams) in the second cohort only.

^3^ Food preference calculated as (HFHS diet consumed) / (CON + HFHS diets consumed).

^4^ Males higher than females.

^5^ Females higher than males.

### Offspring postnatal growth

**Fig 6:**
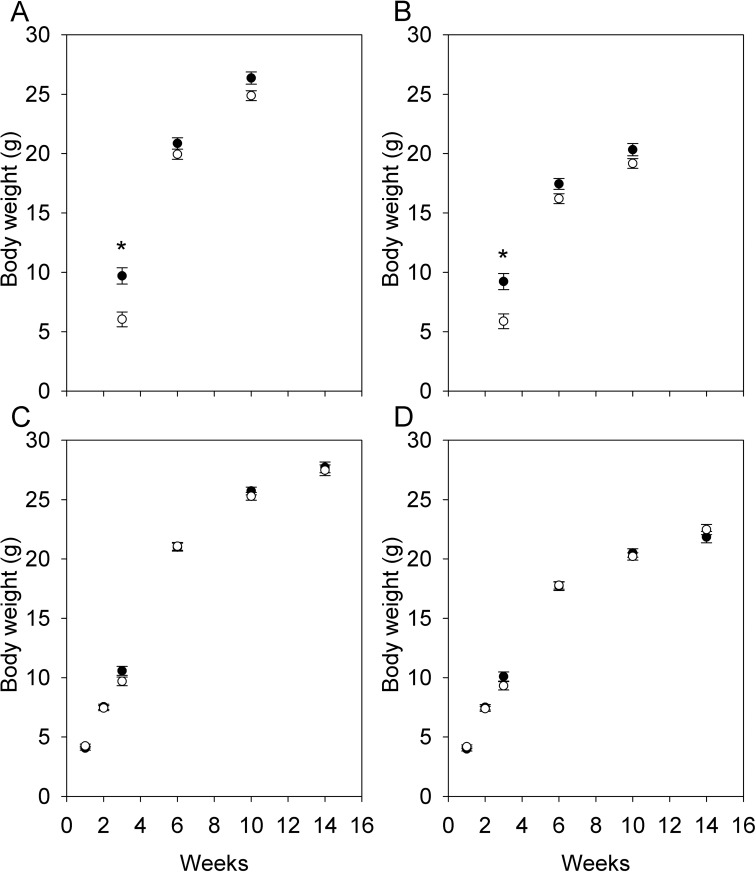
Postnatal growth in offspring. Postnatal growth in males (A, C) and females (B, D) from the first (A, B; N = 5 HFHS dams, 28 pups, 6 CON dams, 43 pups) and second cohorts (C, D; N = 8 HFHS dams, 44 pups, 9 CON dams, 53 pups). Although the diet by sex interaction was not significant at any age, data for each sex are presented separately for clarity, and values are least squares means ± standard error from repeated measures analyses including effects of diet, sex, and the diet by sex interaction with dam as the random subject. Asterisks denote ages at which effect of diet is significant (P < 0.05).

In the first cohort, there was no interaction between sex and maternal diet on postnatal growth at any age (P > 0.4 in all cases). Offspring from HFHS dams were heavier at weaning (3 weeks of age) but not 6 and 10 weeks of age (Fig 6A and 6B). In the second cohort, there was also no interaction between sex and maternal diet on postnatal growth at any age (P > 0.15 in all cases), but in contrast to the first cohort, offspring from HFHS dams were not heavier at 3 weeks of age (Fig 6C and 6D). The diet by cohort interaction was significant at 3 weeks of age (F_1,24_ = 7.87, P = 0.01). In the first cohort, litter size at birth was lower in HFHS dams but we did not standardize litter sizes, and so the difference in mass at weaning could be due to litter size rather than maternal diet. To test this, we added litter size at weaning as a covariate to the analyses of the first cohort, but it was not a significant covariate and the effect of diet remained significant. In the second cohort, body composition at euthanasia (at 19–24 weeks of age) was analysed by QMR. There was no significant interaction between sex and maternal diet for fat or lean mass (P > 0.65 in both cases), and no significant effect of maternal diet for either trait (Table 3).

### Offspring food consumption and metabolic traits

HFHS and CON offspring were all weaned onto the same chow diet. Food consumption at 5–6 weeks of age did not show a significant interaction between sex and maternal diet in either cohort (P > 0.48 in both cases), but food consumption was significantly higher in HFHS offspring in the second cohort and tended to be higher in the first cohort (Table 3). However, in the second cohort, food consumption was also measured in metabolic cages at 18–23 weeks of age, at which point there was no effect of maternal diet (Table 3). At 17–22 weeks of age, offspring were offered both the HFHS and CON experimental diets to assess diet preference, and all mice strongly preferred the HFHS diet (~95% of food consumed by mass was HFHS), but there was no effect of maternal diet, although males had a stronger preference for the HFHS diet than females (Table 3).

**Fig 7:**
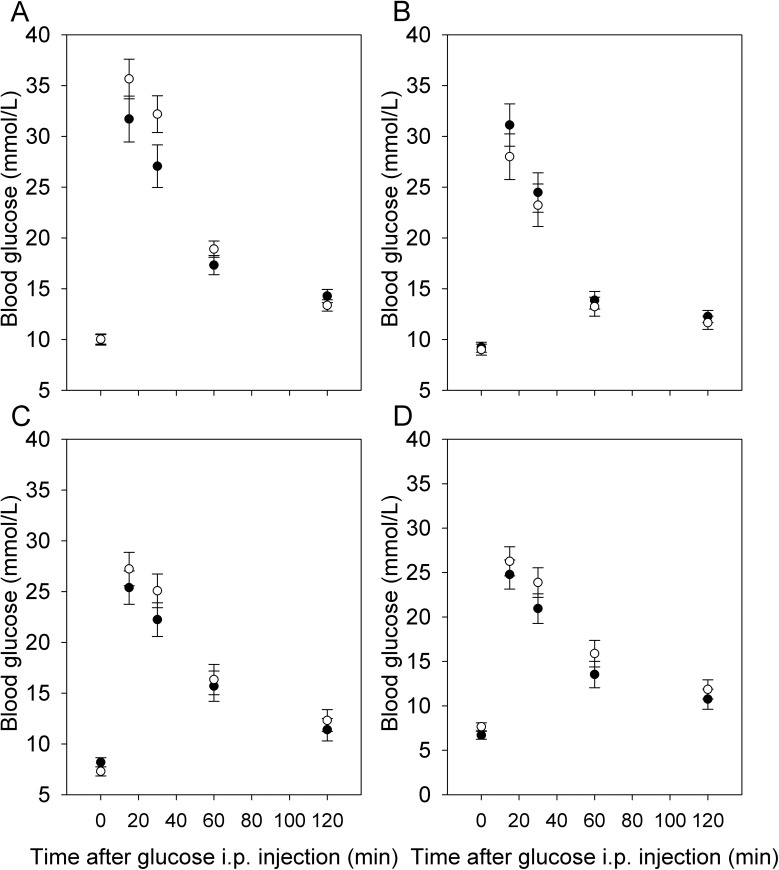
Glucose tolerance in offspring. Glucose tolerance in males (A, C) and females (B, D) from the first (A, B; N = 5 HFHS dams, 24 pups, 6 CON dams, 26 pups) and second (C, D; N = 8 HFHS dams, 16 pups, 9 CON dams, 16 pups) cohorts. Although the diet by sex interaction was not significant, data for each sex are presented separately for clarity, and values are least squares means ± standard error from repeated measures analyses including effects of diet, sex, and the diet by sex interaction with dam as the random subject.

At 15–18 weeks of age, fasting glucose and glucose tolerance, as measured by AUC or piAUC, did not show a significant sex by maternal diet interaction in either cohort (P > 0.05 in all cases). The effect of maternal diet was not significant for any trait in either cohort (P > 0.18 in all cases) (Fig 7). Fasting triglyceride levels were measured in the second cohort only, and did not show a significant sex by maternal diet interaction (F_1,14_ = 1.73, P = 0.21), or an effect of diet (Table 3).

At 18–23 weeks of age, a number of metabolic traits were measured in the second cohort, including food intake, water intake, energy expenditure, oxygen consumption, carbon dioxide production and the respiratory exchange ratio, and none of these traits showed an effect of maternal diet (Table 3) or an interaction between maternal diet and offspring sex (P > 0.05 in all cases).

### Offspring stress response and voluntary locomotor activity

**Fig 8:**
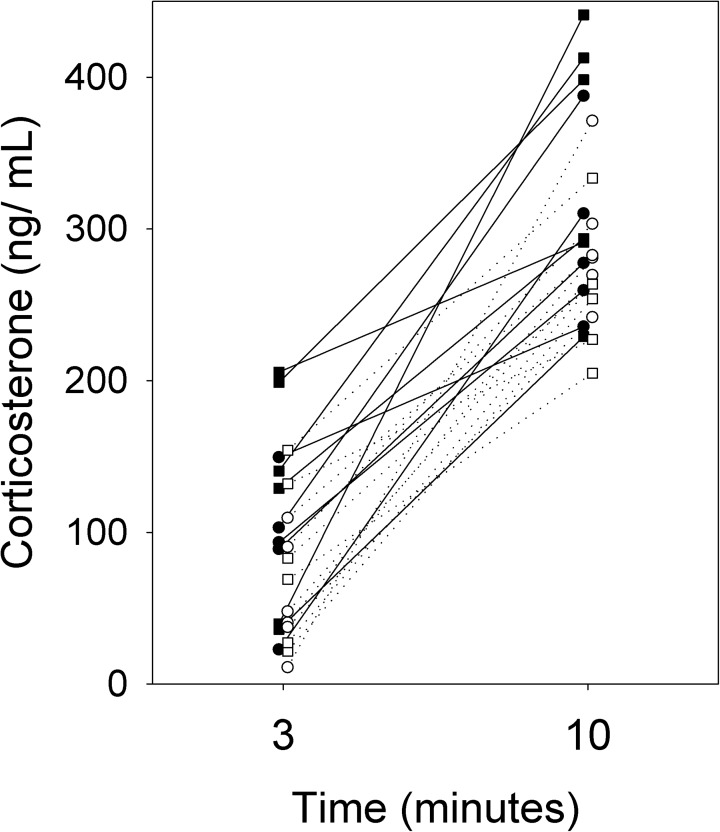
Stress response in offspring. Corticosterone levels after 3 and 10 minutes of restraint stress in HFHS (solid symbols, solid lines) and CON (open symbols, dotted lines) male (squares) and female (circles) offspring from the second cohort (N = 6 HFHS dams, 11 pups, 7 CON dams, 12 pups). Lines connect measurements of individual mice at 3 and 10 minutes of restraint stress.

In the second cohort, the response to stress was assessed in offspring at 9 weeks of age by measuring circulating corticosterone levels after 3 and 10 minutes of restraint stress. In a repeated measures analysis with dam as the random subject, the interactions between maternal diet, sex and time were not significant (P > 0.15 for all), but there was a significant effect of maternal diet, with HFHS offspring having higher circulating corticosterone (F_1,11_ = 5.64, P = 0.04; Fig 8). Circulating corticosterone did not differ between the sexes (F_1,9_ = 1.07, P = 0.33; Fig 8). Consistent with the lack of interaction between diet and time, the difference in corticosterone levels between 3 and 10 minutes did not show an interaction between maternal diet and sex (F_1,8_ = 1.26, P = 0.29), or an effect of diet (F_1,11_ = 0.05, P = 0.83).

Voluntary locomotor activity was measured by use of running wheels over 4 nights in offspring at 16–19 weeks of age. There was no sex by maternal diet interaction and no effect of maternal diet in either cohort (P > 0.1 in all cases). The effect of sex was highly significant in each cohort (P < 0.0001 in each cohort), with females more active than males (Table 3). In the second cohort, spontaneous physical activity (sensu [40]) was measured at 18–23 weeks of age by means of infrared beam breakage by animals moving within metabolic cages. Results were similar to those obtained with running wheel use, i.e., no effect of maternal diet, but females showed greater activity, a difference that was significant in the light phase and marginally non-significant in the dark phase (Table 3).

## Discussion

### Fetal glucocorticoid levels and offspring behaviour

We tested whether a maternal HFHS diet would alter fetal exposure to glucocorticoids and result in programming effects on the behaviour and physiology of adult offspring. We observed a significant interaction between maternal diet and fetal sex on fetal circulating corticosterone, such that HFHS male fetuses had higher plasma corticosterone than CON male fetuses, but there was no effect of maternal diet on female fetuses. Maternal corticosterone was much higher than fetal corticosterone, reflecting the 11β-HSD2 barrier, although maternal and fetal corticosterone levels were significantly correlated, suggesting that the barrier was incomplete [13]. Although we had hypothesized that aberrant fetal exposure would be due to altered placental 11β-HSD expression, we observed no effect of maternal diet on 11β-HSD1 or 11β-HSD2 expression. Elevated corticosterone in male fetuses may therefore have been due to altered 11β-HSD expression earlier in pregnancy, or to altered fetal glucocorticoid production and/or metabolism. A previous study of the same strain of mice fed a diet with a higher fat content found reduced 11β-HSD2 activity in association with elevated maternal corticosterone [21], in contrast to the reduced maternal levels that we observed. Other studies of the same strain of mice have found that a higher fat diet either reduces 11β-HSD2 mRNA expression [22] or has no effect [26]. A maternal low-protein diet did not affect placental 11β-HSD2 expression or activity in mice [41], in contrast to other species.

Despite a sex-specific effect of maternal diet on fetal glucocorticoids, there was little evidence of sex-specific effects on offspring behaviour or physiology. We found that circulating corticosterone levels were higher in offspring of HFHS dams in both sexes at 9 weeks of age, consistent with the increased activity of the offspring HPA axis previously observed in association with altered fetal glucocorticoid exposure [18]. However, this effect was not sex-specific.

### Maternal physiology and pregnancy

Studies of developmental programming use a wide variety of dietary manipulations in different animal models. We do not know of another study to use our diets, and therefore characterized our model in terms of its effects on maternal physiology and frequently studied offspring traits. Females on the HFHS diet were ~15% heavier and had ~45% higher fat mass during pregnancy than control mice on the nutrient-matched CON diet. Whereas maternal glycaemic control is often not reported in such studies [42], we found that HFHS females were hyperglycemic and had impaired glucose tolerance. HFHS females had lower glucocorticoid levels than controls during pregnancy, which has also been observed in obese human pregnancy [43]. In contrast, a previous study of the same line of mice fed a higher fat diet found that it increased maternal levels of corticosterone during pregnancy, and increased the frequency of maternal cannibalism [21]. Despite observing the opposite pattern with respect to maternal glucocorticoid levels, we also observed increased loss of first litters shortly after birth in HFHS females, suggesting that our HFHS diet was a stressor for the female.

HFHS females tended to have higher leptin levels than controls during pregnancy, as previously reported in mice [22,35,44,45] and rats [42], as well as in obese human pregnancy [46]. In contrast to this trend in maternal leptin levels, we found no effect of maternal diet on fetal circulating leptin, consistent with previous observations in sheep and rats [47]. Potentially as a result of lower glucocorticoid and higher leptin levels, HFHS females increased their caloric intake through gestation less than CON females, such that there was no difference in caloric intake and a reduced difference in body mass towards the end of gestation. This behavioural response may have protected fetuses from overnutrition/ overgrowth, avoiding the increase in fetal/ birth weight observed in some studies [35,45].

While leptin and glucocorticoids affect placental function [5,13,46,47], we observed no effect of maternal diet on the placental expression of nutrient transporters or on insulin signaling as measured by levels of placental phosphorylated Akt, although total Akt levels were increased slightly by the HFHS diet. The placenta is thought to integrate maternal and fetal signals to match nutritional supply and demand to the extent possible [24,48] via modulating the expression and activity of nutrient transporters [23,35,45]. We speculate that the mothers’ altered caloric intake was adequate to protect the fetuses from overgrowth and/ or that our dietary manipulation was not sufficiently severe to warrant changes in gene expression. However, the effects of maternal diet on nutrient transporter expression have been found to vary through gestation [22,23,26] and thus we cannot rule out the possibility that nutrient transport was altered earlier in pregnancy.

Inflammatory cytokines are elevated in obese human and rodent pregnancy [6]. A high-fat diet has been observed to elevate circulating IL-6 levels in pregnant mice in some studies [44,49] but not others [35], and we found no such effect.

### Postnatal offspring growth and metabolism

We observed few consistent effects of maternal diet in postnatal offspring. Offspring of HFHS dams were heavier at weaning in the first cohort but not in the second cohort, and there was no difference at 6 weeks of age or older in either cohort. Food consumption was higher in HFHS offspring at 5–6 weeks of age, but not in older mice. Fasting glucose, glucose tolerance and other metabolic traits were not affected by maternal diet in either cohort. While the programming effects of maternal overnutrition have received substantial attention, a lack of effect of a maternal high-fat diet on offspring food consumption, post-weaning body weight, and glycaemic control is not unusual [42,50,51]. In some cases a postnatal metabolic challenge is required to reveal programming effects [50], and potentially we would have observed more substantial programming effects had we weaned offspring onto the HFHS diet.

## Conclusions

To our knowledge, ours is the first study to examine the effects of a maternal high-fat, high-sucrose diet on fetal glucocorticoid levels. We found a sex-specific effect of maternal diet on fetal glucocorticoid levels, but little evidence of programming consequences of this diet for adult offspring. Offspring of HFHS dams had higher circulating corticosterone as young adults, but this effect was not sex-specific. Although sex-specific programming effects are frequently reported in the literature, few studies explicitly test for a sex by diet interaction as we did [38].

## Supporting information

S1 File Data underlying findings. Raw data used in analyses.
(XLSX)
